# Primary spindle cell sarcoma of gallbladder: An unusual case report and a literature review

**DOI:** 10.1097/MD.0000000000028549

**Published:** 2022-01-14

**Authors:** Xin Long, Yan Chen, Wei-Xun Chen, Yu Wu, Jia Song, Jin Chen, Lei Zhang

**Affiliations:** a Hepatic Surgery Center, Institute of Hepato-Pancreato-Biliary Surgery, Tongji Hospital, Tongji Medical College, Huazhong University of Science and Technology, Wuhan, China; b Department of Pediatrics, Union Hospital, Tongji Medical College, Huazhong University of Science and Technology, Wuhan, Hubei Province, China; c Department of Hepatobiliary Surgery, Shanxi Bethune Hospital, Shanxi Academy of Medical Sciences, Shanxi Medical University; Shanxi Tongji Hospital, Tongji Medical College, Huazhong University of Science and Technology, Taiyuan, China.

**Keywords:** diagnosis, gallbladder, gallbladder carcinoma, primary spindle cell sarcoma, surgical treatment

## Abstract

**Introduction:**

Primary spindle cell sarcoma of the gallbladder is a rare condition.

**Patient concerns:**

A 67-year-old woman was admitted to a local hospital with a chief complaint of abdominal pain in the right upper quadrant for the past 2 months.

**Diagnosis and intervention:**

Surgical resection was performed following the diagnosis of primary gallbladder sarcoma with local hepatic metastasis. Histological examination confirmed a diagnosis of primary spindle cell sarcoma and hepatic metastasis with simultaneous cholecystolithiasis.

**Outcomes:**

Adjuvant chemoradiation therapy was not performed because the patient refused treatment. Three months after the surgery, a relapsed lesion was diagnosed. The patient underwent transcatheter arterial chemoembolization.

**Conclusions:**

The disease should be differentially diagnosed from gallbladder carcinoma or carcinosarcoma with hepatic metastasis. An aggressive surgical approach should be based on a balance between the risk of surgery and the outcome.

## 1 Introduction

Soft tissue sarcomas are rare solid tumors of mesenchymal cell origin that exhibit a heterogeneous mix of clinical and pathological characteristics. They account for 1% of adult cancers and 10% to 20% of adolescent and young adult cancers.^[1]^ With the widespread use of regular medical checkups and improvements in early diagnosis, the occurrence of primary sarcoma of the gallbladder is becoming more frequent^[2]^; however, knowledge concerning the clinical features and outcomes of primary sarcoma of the gallbladder remains limited. Here, we report a case of primary spindle cell sarcoma with hepatic metastasis.

## 2 Case presentation

A 67-year-old woman was admitted to a local hospital with a chief complaint of abdominal pain in the right upper quadrant for the past two months. A multidetector computed tomography (CT) scan of the abdomen showed a thickened gallbladder wall with gallbladder stones and multiple hypodense tumorous lesions adjacent to the gallbladder in the lower part of segments IV and V of the liver. Emission computed tomography revealed no bone metastases. A biopsy of the liver lesion was performed at a local hospital. Histopathological examination revealed the possibility of a metastatic inflammatory myofibroblastic tumor originating from the gallbladder. The patient was then transferred to our hospital for further diagnosis and treatment.

The patient had a history of high blood pressure for 2 years: which was controlled by oral administration of amlodipine and telmisartan. There was no remarkable family, medical, or genetic history. The patient was in good general health and had undergone a significant weight loss. Her vital signs (heart rate, respiration rate, blood pressure, and body temperature) were within the normal limits. No positive signs were observed during physical examination. The complete blood count and serum biochemistry data on admission were normal. The patient also showed normal levels of tumor markers, including alpha-fetoprotein (AFP: 0.87 ng/ml, normal: ≤7 ng/ml), carcinoembryonic antigen (CEA: 2.09 ng/ml, normal: ≤5.0 ng/ml), and carbohydrate antigen19-9 (CA19-9: 0.90 U/ml, normal: ≤34 U/ml).

**Figure 1:**
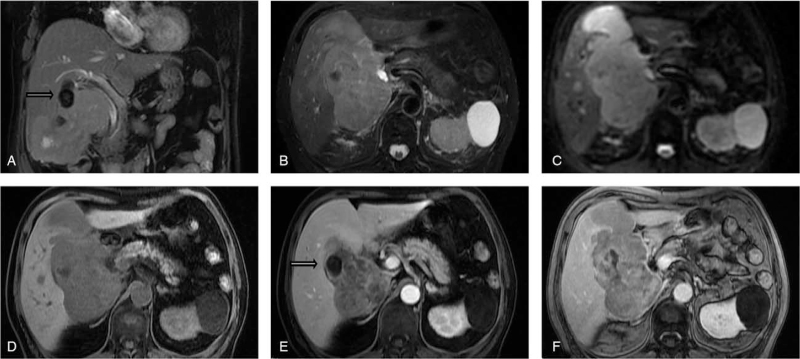
Abdominal magnetic resonance imaging with perfusion-weighted imaging showing a 13.5 × 9.9 cm enlarged gallbladder with gallbladder stones (the largest measured 3.5 × 2.1 cm) and thickened wall, and invasion of surrounding liver. A and B: T2-weighted images, C: DWI image, D: water + c phase, E: water phase, F: out of phase image, and arrow: gallbladder stones. DWI = dippusion-weighted imaging.

**Figure 2:**
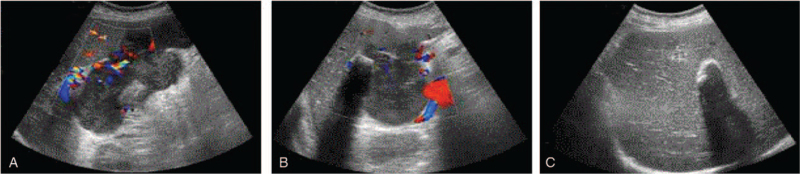
Abdominal ultrasound demonstrating enlarged gallbladder with destroyed structure (A), filled with hypoechoic mass (B) and gallbladder stones (C).

Magnetic resonance imaging (MRI) with perfusion-weighted imaging confirmed the presence of gallbladder stones (the largest measured 3.5 × 2.1 cm) and thickened wall in the enlarged gallbladder (13.5 × 9.9 cm), and hypervascular tumors in the liver (Fig. 1A–F). The abdominal ultrasonography data were consistent with these data (Fig. 2A–C). Thus, the preoperative diagnosis was a primary sarcoma of the gallbladder with hepatic metastasis. A multidisciplinary team (MDT) was composed of a variety of specialists who each brought a perspective to the holistic management of this patient, including surgeons, medical oncologists, radiation oncologists, pathologists, radiologists, gastroenterologists, and ultrasonologists. After the MDT discussion, the decision was made to remove the gallbladder and invaded liver tissues.

The patient was informed of the risks involved with the surgery before consent for the operation was obtained. After preoperative preparation, the patient underwent exploratory laparotomy. During laparotomy, the gallbladder was enlarged, showed wall thickening, and had numerous stones (the largest measuring 6 × 6 cm). Exploration also indicated an 8 × 6 cm rigid lesion fused by multiple masses in liver segments IVb and V. All of these formed a mass of 14 × 10 cm. In addition, the tumor invaded the serosa of the colon transversum and duodenal bulb, as well as the pancreatic capsule. Enlarged lymph nodes were not observed. The patient underwent cholecystectomy, resection of liver segments IV and V, and regional lymphadenectomy.

**Figure 3:**
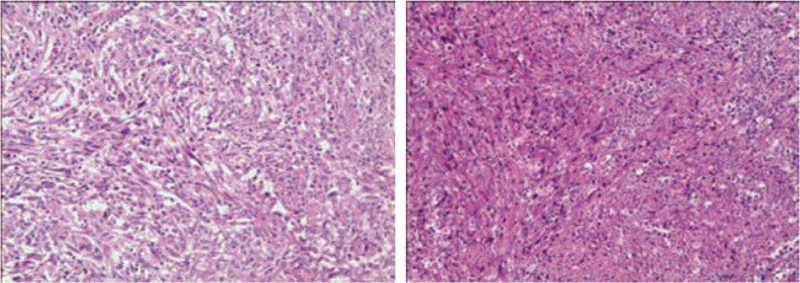
Histopathological examination of resected specimen (hematoxylin–eosin staining, magnification 100×).

Postoperative histopathological examination confirmed primary spindle cell sarcoma and hepatic metastasis, with simultaneous cholecystolithiasis (Fig. 3), which included heterogeneous, moderately differentiated features, most probably originating from fibrogenic or myofibrogenic sources, with an organizational structure akin to an inflammatory myofibroblastic tumor with focal necrosis. The lymphoid nodes in groups 12a, 12b, 12c, 8, and 13 showed no neoplastic cells. Immunohistochemical examination revealed positive expression of SDHB, cyclin D1, S-100 (focal), β-catenin (cytoplasmic), and MDM2 (in part). The tumor was negative for CD 163, CD68, PCK, ALK, ROS1, CD117, CD34, DOG1, SMA, DES, Caldsmon, SOX100, MSA, EMA, CK18/8, CK19, CD1α, CD21, CD23, CD35, langerin, CD45-LCA, HMB45, Melan-A, Braf (V600E), and E-cadherin. Molecular pathology showed that EBER FISH was negative.

After 10 days of recovery, the patient was discharged without any complications. Adjuvant chemoradiation therapy was not administered because of the patient's refusal to consent. Three months after the surgery, the patient underwent ultrasonography and MRI. Both examinations implied that there was a relapsed lesion (4 × 5 cm) in segment VIII. The patient underwent the treatment involving transcatheter arterial chemoembolization. The patient was discharged after 4 days. In the 11-month period since the second intervention, the patient was asymptomatic and had satisfactory general health, as confirmed by telephone interviews at 2-month intervals. More detailed information about the patient could not be obtained because the patient refused to consent to new imaging tests.

## 3 Discussion and conclusions

Primary sarcoma of the gallbladder is a rare form of tumor compared with other malignancies of the gallbladder and represents approximately 1.5% of all malignant gallbladder diseases.^[3]^ A variety of tumors composed of sarcomatous elements include carcinosarcoma and 11 types of primary sarcoma (extraskeletal osteosarcoma, Langerhans cell sarcoma, synovial cell sarcoma, rhabdomyosarcoma, malignant fibrous histiocytoma variants, leiomyosarcoma, angiosarcoma, neurofibrosarcoma, myogenous sarcoma, and liposarcoma) (Tables 1 and 2). To date, there have been 65 cases reported in English outlets.^[4–22]^Table 1 presents the results.

**Table 1:** Literature review of primary sarcomas occurring in gallbladder.

Cases	Case number	Age (yrs)/sex	Gallbladder stones	Location of tumor in liver	Histopathological examination	Treatment	Prognosis (mo^∗^)
Perez-Montiel D et al^[4]^	1	34/F	With	No	LMS	Laparotomy	24/Alive
Olgyai G et al^[5]^	1	61/F	With	No	EOS	Laparotomy	9/Death
Shankar A et al^[6]^	1	45/F	With	No	EOS	Laparotomy	12/Alive
Paasch C et al^[7]^	24	66.8 ± 15.3; M/F:6/18	With (12) Without (12)	4: SIV, SIV/V, SIV/V, N/A	LMS	Laparotomy:15 Chemotherapy:1 Drainage: 1	Alive (8): 3-30 Death (6): 1.9 ± 2.1 N/A (10)
García Marín A et al^[8]^	2	79/M 81/F	N/A	Bilobar SIV/V	LMS	Laparotomy	Death: 0.03, 0.23
Zhao G et al^[9]^	1	74/F	Without	No	LCS	Laparotomy	8/Alive
Bader H et al^[10]^	1	79/M	Without	No	liposarcoma	Laparotomy	24/Death
Hamada T et al^[11]^	1	49/F	Without	No	liposarcoma	3 laparotomy	42/Alive
Ma Y et al^[12]^	1	70/F	Without	No	liposarcoma	Laparotomy	N/A
da Costa AC et al^[13]^	1	71/F	With	No	liposarcoma	Laparotomy	8/Alive
Al-Daraji WI et al^[14]^	15 A: 12; C: 3	A: 65.3 ± 19.2 C: 1.5, 1.8, 3; M/F: 4/11	With (10) Without (5)	N/A	MFHV: 8 LMS: 2 RMS:3 children AS: 2	Laparotomy: 15 Chemotherapy: 3 Radiation: 2	Alive (2)^†^: 132,324 Death (10): 15.5 ± 20.9; N/A (3)
Liu XF et al^[15]^	1	72/F	Without	No	neurofibrosarcoma	Laparotomy	N/A
Odashiro AN et al^[16]^	7	67.4 ± 13.0; M/F: 4/2	With (5)	N/A	AS	Laparotomy	Alive (1): 60 Death (6): 1.8 ± 1.5
Costantini R et al^[17]^	1	57/M	With	No	AS	Laparotomy	60/Alive
Sánchez Acedo P et al^[18]^	1	81/M	Without	SV	AS	Laparotomy	0.67/Death
Husain EA et al^[19]^	7	72.1 ± 7.4; M/F: 1/6	Most	N/A	MFHV: 3 LMS: 1 AS: 1 Liposarcoma: 2	Laparotomy: 7 Chemotherapy: 3	All death:1.5-24
Qayum A et al^[20]^	1	51/M	Without	No	SCS	Laparotomy and radiation	N/A
Nestler G et al^[21]^	1	66/M	Without	No	Myogenous sarcoma	2 laparotomy	11/Death
Gahlot GP et al^[22]^	1	72/F	With	No	EOS	Laparotomy	N/A
Present case	1	67/F	With	SIV/V	Spindle cell sarcoma	Laparotomy	12/Alive

A = adult, As = angiosarcoma, C = children, EOS = extraskeletal osteosarcoma, F = female, LCS = Langerhans cell sarcoma, LMS = leiomyosarcoma, M = male, MFHV = malignant fibrous histiocytoma variants, RMS = rhabdomyosarcoma, S = segment, SCS = synovial cell sarcoma.

∗ Thirty days in 1 month.

† Both were children with RMS, the other was lost to follow-up.

**Table 2:** Summary of primary sarcomas occurring in gallbladder.

Pathological type	n	Age (yrs)	Sex (M/F)	With gallbladder stones (%)	Liver metastasis or invasion (%)	Treatment	Prognosis (mo^∗^)
EOS	3	45,61,72	0/3	2/3 (66.7%)	0/3 (0.0%)	Laparotomy	Death (1): 8; Alive (1): 12; N/A (1)
LCS	1	74	0/1	0 (0.0%)	0 (0.0%)	Laparotomy	Alive: 8
Neurofibrosarcoma	1	72	0/1	0 (0.0%)	0 (0.0%)	Laparotomy	N/A
SCS	1	51	0/1	0 (0.0%)	0 (0.0%)	Laparotomy and radiation	N/A
Myogenous sarcoma	1	66	1/0	0 (0.0%)	0 (0.0%)	Laparotomy	Death: 11
RMS	3	1.5, 1.8, 3	1/2	0 (0.0%)	0 (0.0%)	Laparotomy: 3 Chemotherapy: 2 Radiation: 1	Alive (2): 132,324; N/A (1)
Liposarcoma	6	67.2 ± 10.1	1/5	1/4 (25.0%)	0/4 (0.0%)	Laparotomy: 6 Chemotherapy: 1	Alive (2): 8,42; Death (3): 24, N/A (2); N/A (1)
MFHV	11	69.1 ± 16.7	4/7	Most	N/A	Laparotomy: 11 Chemotherapy: 2 Radiation: 1	Death (11): 20.3 ± 26.0, N/A (5)
LMS	28	65.6 ± 17.0	6/22	Most	6/28 (21.4%)	Laparotomy: 28 Chemotherapy: 2 Radiation: 1	Alive (8): 3–30; Death (11): 5.1 ± 6.9, N/A (4); N/A (9)
AS	12	67.2 ± 12.3	6/6	Most	1/12 (8.3%)	Laparotomy	Alive (2):60,60 Death (10): 2.2 ± 2.2, N/A (1)
Spindle cell srcoma	1	67	0/1	With	1/1 (100%)	Laparotomy	Alive: 12
Total^†^							
11	65	66.4 ± 14.7^∗^	19/46	34/57 (59.6%)	8/47 (17.0%)		Alive (16): 3–60;Death (37): 8.8 ± 14.7, N/A (12); N/A (12)

As = angiosarcoma, EOS = extraskeletal osteosarcoma, F = female, LCS = Langerhans cell sarcoma, LMS = leiomyosarcoma, M = male, MFHV = malignant fibrous histiocytoma variants, RMS = rhabdomyosarcoma, and SCS = synovial cell sarcoma.

∗ Different age group: ≤30: 1 (1.54%); 31∼: 4 (6.15%); 41∼: 4 (6.15%); 51∼: 9 (13.8%); 61∼: 18 (27.7%); 71∼: 16 (24.6%); 81∼: 12 (18.5%); 91∼: 1 (1.54%).

† Not including three children.

According to the World Health Organization classification of soft tissue and bone tumors, there are more than 50 different histologic subtypes of sarcomas.^[23]^ Nearly one-quarter of these types have been found in primary sarcomas of the gallbladder. Based on our data, this was more frequent in women with an M/F ratio of 1:2.42. It occurred at all ages, from 1.5 years to 91 years, with the highest frequency occurring between the ages of 50 and 90 years (approximately 84.6% of all cases). Gallstones are invariably present in several types of primary sarcomas of the gallbladder (Tables 1 and 2), and the presenting symptoms are those of chronic cholecystitis.^[4–22]^

In this series of 65 cases reviewed in the literature, no case was correctly diagnosed before surgery, except when it was preoperatively diagnosed by biopsy. CT and MRI are considered to be the best diagnostic techniques for cancer staging and surgical strategy guidance. However, the data indicate that in sarcomas, the rate of diagnostic inaccuracy ranges between 20% and 30%.^[24]^ The reasons for this may be

1) low incidence and lack of experience,
2) lack of obvious symptoms and typical radiological features, and
3) non-specific tumor markers.

**Figure 4:**
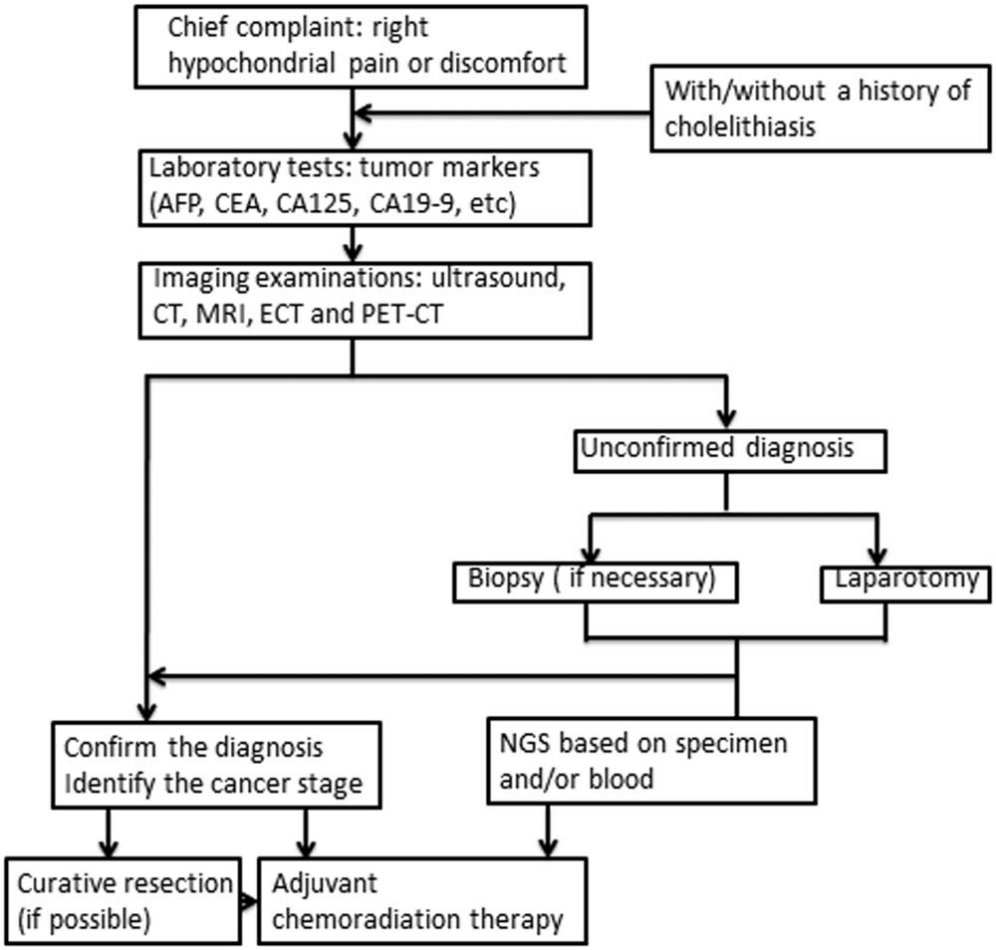
Clinical algorithms for the evaluation of primary sarcoma of gallbladder. AFP = alpha-fetoprotein, CA125 = carbohydrate antigen125, CEA = carcinoembryonic antigen, CT = computed tomography, ECT = emission computed tomography, MRI = magnetic resonance imaging, NGS = next-generation sequencing, and PET-CT = positron emission computerized tomography.

Apart from these cases, it might also have been due to the intrinsic complexity of sarcoma, technological complexity, and the lower influence of educational efforts.^[24]^ We considered it difficult to differentiate between primary sarcoma, carcinosarcoma, and carcinoma of the gallbladder; therefore, we searched the literature and listed the main differences between the most common tumors of the gallbladder in Table 3.^[25–29]^ Recently, studies have shown that positron emission computed tomography scanning is useful in differentiating malignant from benign diseases when using various radiopharmaceutical agents to improve the detection and characterization of the pathophysiology of the disease.^[30]^ The fusion of positron emission computed tomography-acquired images with CT scans or MRI has significantly improved the overall diagnostic accuracy for sarcoma.^[31]^ Histopathology from biopsy or liver resection remains the gold diagnostic standard.^[32]^ If the lesions can be surgically resected, histological examination of the whole lesion is more reliable than a biopsy because insufficient, missed, or ample necrotic tissues may not be representative of the entire lesion.^[33]^ Needle-tract implantation and complications (such as prolonged internal bleeding, bile leakage, and infection) are other reasons why preoperative biopsy of the liver is not recommended.^[34]^ The detailed diagnostic procedure is shown in Figure 4.

**Table 3:** Differential diagnosis between primary sarcomas of gallbladder, carcinosarcoma of gallbladder and gallbladder carcinoma with hepatic metastasis.

	PSG	CG^[25–28]^	GC with HM^[29]^
Incidences	Rare, 65 cases (no 3 children)	Rare, about 100 cases, <1% of gallbladder cancer	Geography/ethnicity (1.1–27/10^5^)
Gender	Major in F (F/M = 2.4/1)	Major in F (F/M = 4.3/1)	Major in F (F/M = 2–6/1)
Age (yrs)	Old age (Mean = 68.8)	Old age (Mean = 68.8)	Old age (mean = 67)
HBV/HCV	Partial patients	N/A	Not
Gallbladder stones	50%	33–75%	∼85%
AFP	Not	Not	Not
CEA	Not	Not	Elevated
CA19-9	Not	Not	Elevated
Number of liver masses	Simple or multiple	Simple or multiple	Multiple
Location of liver masses	Any segments in the liver	Most adjacent to the gallbladder	Adjacent to the gallbladder
Invasion of GC	Not found	Often	Presentation
Imaging manifestation	Hypovascular or hypervascular	Hypervascular	Hypovascular

AFP = alpha-fetoprotein, CA19-9 = carbohydrate antigen 19-9, CEA = carcinoembryonic antigen, CG = carcinosarcoma of gallbladder, F = female, GC = gallbladder carcinoma, HBV = hepatitis B virus, HCV = hepatitis C virus, HM = hepatic metastasis, M = male, and PSG = primary sarcomas of gallbladder.

MDT in oncology is defined as cooperation between different specialized professionals involved in cancer care with the overarching goal of improving treatment efficiency and patient care. MDT discussion is conducive to prompt diagnosis and rapid referral, and provides patient-centered, specialized, integrated multidisciplinary care and treatment.^[35]^ Based on the suggestions from the MDT, we decided to implement surgical therapy because curative resection, if possible, most effectively prolongs patient survival. Most of the patients with sarcoma of the gallbladder underwent surgical treatment, and several were further treated with adjuvant chemotherapy and/or radiation (Tables 1 and 2); however, the prognosis was not satisfactory; more than half of the patients died within 8.8 ± 14.7 months after surgery, which might be higher due to partial loss of follow-up opportunities, making this a concern for surgeons and oncologists about how to treat patients with this rare disease.

The pathogenesis of sarcoma of the gallbladder has not been clarified, and some factors, such as germline mutations, radiation, and environmental exposure, are involved.^[36]^ Gallbladder stones and/or infectious agents cause cancer as a result of recurrent trauma and chronic inflammation.^[37]^ In our study, 59.6% of patients were diagnosed with cholecystolithiasis, but it remains unclear whether gallbladder stones and concomitant inflammation initiate the transformation of mesenchymal cells. Many studies have shown that sarcoma might originate from mesenchymal stem cells (MSCs), which show that rodent MSCs are prone to spontaneous sarcomagenesis upon prolonged in vitro culture, and that MSCs have been used to establish several crucial experimental sarcoma models.^[38]^ The rarity of the disease means that clinical data are limited; therefore, the detailed etiology and mechanism still need further clarification. Recently, next-generation sequencing (NGS) has been recommended for application in oncology, spanning from diagnostics to prognosis and from prevention to treatability.^[39]^ NGS techniques have provided additional insights into the genetic diversity and complexity of sarcomas, including the potential therapeutic implications of some genetic alterations.^[40]^ In this study, the patients refused NGS and adjuvant chemotherapy because of economic limitations and doubts regarding adverse reactions to chemotherapy.

In conclusion, primary sarcomas of the gallbladder are rare. Owing to the lack of apparent radiological features, it is not easily differentiated from other types of gallbladder tumors. Therefore, it is necessary to develop more accurate diagnostic techniques and administer more refined treatment strategies to diagnose and treat this disease.

## Acknowledgments

The authors thank MD Chao-Hong Yu for his assistance with language checking and revision.

## Author contributions

LX, CY and ZL designed the study and wrote the manuscript. CWX, WY, SJ, and CJ participated in study design, data collection, and drafting of the manuscript. LX, CY, and ZL participated in language checking. All authors have read and approved the manuscript.

**Conceptualization:** Xin Long, Yan Chen, Lei Zhang.

**Data curation:** Jia Song.

**Formal analysis:** Jin Chen.

**Methodology:** Wei-Xun Chen, Yu Wu, Jia Song.

**Supervision:** Lei Zhang.

**Writing – original draft:** Xin Long, Yan Chen, Lei Zhang.

**Writing – review & editing:** Lei Zhang.

## References

[1] Poon E Quek R. Soft tissue sarcoma in Asia. Chin Clin Oncol 2018;7:46. 30173536 10.21037/cco.2018.08.06

[2] von Mehren M Randall RL Benjamin RS. Soft tissue sarcoma, version 2.2018, NCCN clinical practice guidelines in oncology. J Natl Compr Canc Netw 2018;16:536–63. 29752328 10.6004/jnccn.2018.0025

[3] Gamboa AC Gronchi A Cardona K. Soft-tissue sarcoma in adults: an update on the current state of histiotype-specific management in an era of personalized medicine. CA Cancer J Clin 2020;70:200–29. 32275330 10.3322/caac.21605

[4] Perez-Montiel D Mucientes F Spencer L Klaassen R Suster S. Polypoid leiomyosarcoma of the gallbladder: study of a case associated with adenomyomatoushyperplasia. Ann Diagn Pathol 2004;8:358–63. 15614741 10.1053/j.anndiagpath.2004.08.006

[5] Olgyai G Horváth V Banga P Kocsis J Buza N Oláh A. Extraskeletal osteosarcoma located to the gallbladder. HPB (Oxford) 2006;8:65–6. 18333242 10.1080/13651820600573204 PMC2131365

[6] Shankar A Sahoo RK Malik A Kakkar A Kishor Rath G. Extra skeletal osteosarcoma of gall bladder: a case report. J Egypt Natl Canc Inst 2015;27:231–4. 26077931 10.1016/j.jnci.2015.05.002

[7] Paasch C Salak M Mairinger T Theissig F. Leiomyosarcoma of the gallbladder – a case report and a review of literature. Int J Surg Case Rep 2020;66:182–6. 31864148 10.1016/j.ijscr.2019.11.062 PMC6928315

[8] García Marín A Bernardos García L Martín Gil J de Colsa DS Fuentes FT. Primary leiomyosarcoma of the gallbladder. Rev Esp Enferm Dig 2010;102:67–8. 20187695 10.4321/s1130-01082010000100019

[9] Zhao G Luo M Wu ZY. Langerhans cell sarcoma involving gallbladder and peritoneal lymph nodes: a case report. Int J Surg Pathol 2009;17:347–53. 18805873 10.1177/1066896908324128

[10] Bader H Vallon H. Liposarcoma of the gallbladder and the peritoneum. A case report. Zentralbl Allg Pathol 1983;127:45–9. 6858427

[11] Hamada T Yamagiwa K Okanami Y. Primary liposarcoma of gallbladder diagnosed by preoperative imagings: a case report and review of literature. World J Gastroenterol 2006;12:1472–5. 16552824 10.3748/wjg.v12.i9.1472 PMC4124333

[12] Ma Y Wei S Peker D. An extremely rare primary gallbladder myxoid liposarcoma associated with amplification of DDIT3 gene. J Gastrointestin Liver Dis 2014;23:460–1. 25532011

[13] da Costa AC Santa-Cruz F Sena BF. Dedifferentiated liposarcoma of the gallbladder: first reported case. World J Surg Oncol 2018;16:221. 30419915 10.1186/s12957-018-1520-5 PMC6233360

[14] Al-Daraji WI Makhlouf HR Miettinen M. Primary gallbladder sarcoma: a clinicopathologic study of 15 cases, heterogeneous sarcomas with poor outcome, except pediatric botryoid rhabdomyosarcoma. Am J Surg Pathol 2009;33:826–34. 19194282 10.1097/PAS.0b013e3181937bb3

[15] Liu XF Tang K Sui LL Xu G. Neurofibrosarcoma of the gallbladder: a case report. World J Surg Oncol 2013;11:189. 23938157 10.1186/1477-7819-11-189 PMC3765290

[16] Odashiro AN Pereira PR Odashiro Miiji LN Nguyen GK. Angiosarcoma of the gallbladder: case report and review of the literature. Can J Gastroenterol 2005;19:257–9. 15861270 10.1155/2005/794869

[17] Costantini R Di Bartolomeo N Francomano F Angelucci D Innocenti P. Epithelioid angiosarcoma of the gallbladder: case report. J Gastrointest Surg 2005;9:822–5. 15985238 10.1016/j.gassur.2005.01.286

[18] Sánchez Acedo P Herrera Cabezón J Tarifa Castilla A Zazpe Ripa C Lera Tricas JM. Epithelioid angiosarcoma of the gallbladder. Case report and review of the literature. An Sist Sanit Navar 2015;38:333–7. 26486544 10.23938/ASSN.0085

[19] Husain EA Prescott RJ Haider SA. Gallbladder sarcoma: a clinicopathological study of seven cases from the UK and Austria with emphasis on morphological subtypes. Dig Dis Sci 2009;54:395–400. 18618258 10.1007/s10620-008-0358-z

[20] Qayum A Riall TS Srinivasan R Salazar FA. A patient with synovial cell sarcoma primary to the gallbladder and common bile duct. J Gastrointest Surg 2008;12:609–11. 17768664 10.1007/s11605-007-0260-6

[21] Nestler G Halloul Z Evert M Dombrowski F Lippert H Meyer F. Myogenous sarcoma of the gallbladder with a hemangiopericytomatous pattern. J Hepatobiliary Pancreat Surg 2007;14:197–9. 17384914 10.1007/s00534-006-1130-4

[22] Gahlot GP Roy M Jain H. Primary extraskeletal osteosarcoma of gall bladder. Trop Gastroenterol 2015;36:277–80. 27509713 10.7869/tg.309

[23] WHO Classification of Tumours Editorial Board. WHO Classification of Tumours of Soft Tissue and Bone. 5th ed. Lyon, France: IARC Press; 2020.

[24] Sbaraglia M Bellan E Dei Tos AP. The 2020 WHO classification of soft tissue tumours: news and perspectives. Pathologica 2020;DOI 10.32074/1591-951X-213. [Online ahead of print]. 10.32074/1591-951X-213 PMC8167394 33179614

[25] Faujdar M Gupta S Jain R Wadhwani D. Carcinosarcoma of the gallbladder with heterologous differentiation: a case report. J Gastrointest Cancer 2015;46:175–7. 25698405 10.1007/s12029-015-9692-5

[26] Kim HH Hur YH Jeong EH. Carcinosarcoma of the gallbladder: report of two cases. Surg Today 2012;42:670–5. 22391981 10.1007/s00595-012-0160-6

[27] Wada Y Takami Y Tateishi M. Carcinosarcoma of the gallbladder: report of a case. Clin J Gastroenterol 2014;7:455–9. 26184028 10.1007/s12328-014-0522-2

[28] Okabayashi T Sun ZL Montgomey RA Hanazaki K. Surgical outcome of carcinosarcoma of the gall bladder: a review. World J Gastroenterol 2009;15:4877–82. 19842216 10.3748/wjg.15.4877 PMC2764963

[29] Roa JC Basturk O Adsay V. Dysplasia and carcinoma of the gallbladder: pathologic evaluation, sampling, differential diagnosis and clinical implications. Histopathology 2021;DOI 10.1111/his.14360. [Online ahead of print]. 10.1111/his.14360 33629395

[30] Igrec J Fuchsjäger MH. Imaging of bone sarcomas and soft-tissue sarcomas. Rofo 2021;DOI 10.1055/a-1401-0215. [Online ahead of print]. 10.1055/a-1401-0215 33772487

[31] Behzadi AH Raza SI Carrino JA. Applications of PET/CT and PET/MR Imaging in Primary Bone Malignancies. PET Clin 2018;13:623–34. 30219192 10.1016/j.cpet.2018.05.012 PMC7466825

[32] Bourcier K Le Cesne A Tselikas L. Basic knowledge in soft tissue sarcoma. Cardiovasc Intervent Radiol 2019;42:1255–61. 31236647 10.1007/s00270-019-02259-w

[33] Bret PM Labadie M Bretagnolle M Paliard P Fond A Valette PJ. Hepatocellular carcinoma: diagnosis by percutaneous fine needle biopsy. Gastrointest Radiol 1988;13:253–5. 2838372 10.1007/BF01889073

[34] Kim SH Lim HK Lee WJ Cho JM Jang HJ. Needle-tract implantation in hepatocellular carcinoma: frequency and CT findings after biopsy with a 19.5-gauge automated biopsy gun. Abdom Imaging 2000;25:246–50. 10823443 10.1007/s002610000025

[35] Selby P Popescu R Lawler M Butcher H Costa A. The value and future developments of multidisciplinary team cancer care. Am Soc Clin Oncol Educ Book 2019;39:332–40. 31099640 10.1200/EDBK_236857

[36] Popovich JR Kashyap S Cassaro S. Sarcoma. 2020. StatPearls [Internet]. Treasure Island (FL): StatPearls Publishing; 2021.

[37] Huang D Joo H Song N Cho S Kim W Shin A. Association between gallstones and the risk of biliary tract cancer: a systematic review and meta-analysis. Epidemiol Health 2021;e2021011. DOI 10.4178/epih.e2021011. 10.4178/epih.e2021011 PMC8060519 33541011

[38] Hatina J Kripnerova M Houfkova K. Sarcoma stem cell heterogeneity. Adv Exp Med Biol 2019;1123:95–118. 31016597 10.1007/978-3-030-11096-3_7

[39] Morganti S Tarantino P Ferraro E D’Amico P Duso BA Curigliano G. Next generation sequencing (NGS): a revolutionary technology in pharmacogenomics and personalized medicine in cancer. Adv Exp Med Biol 2019;1168:09–30. 10.1007/978-3-030-24100-1_2 31713162

[40] Italiano A. Is there value in molecular profiling of soft-tissue sarcoma? Curr Treat Options Oncol 2018;19:78. 30523434 10.1007/s11864-018-0589-y

